# Comprehensive analysis of transcriptome and pathway interactions in periodontitis

**DOI:** 10.1590/1678-7757-2025-0396

**Published:** 2025-10-20

**Authors:** Bruno César de Vasconcelos GURGEL, Nathalia VILELA, Kaio Henrique SOARES, Luiz Eduardo Rodrigues JULIASSE, Ikramuddin AUKHIL, Poliana Mendes DUARTE

**Affiliations:** 1 Universidade Federal do Rio Grande do Norte Departamento de Odontologia Natal RN Brasil Universidade Federal do Rio Grande do Norte, Departamento de Odontologia, Natal, RN, Brasil.; 2 Universidade de São Paulo Faculdade de Odontologia, Departamento de Estomatologia Divisão de Periodontia São Paulo SP Brasil Universidade de São Paulo, Faculdade de Odontologia, Departamento de Estomatologia, Divisão de Periodontia, São Paulo, SP, Brasil.; 3 Universidade Federal dos Vales do Jequitinhonha e Mucuri Departamento de Odontologia Diamantina MG Brasil Universidade Federal dos Vales do Jequitinhonha e Mucuri, Departamento de Odontologia, Diamantina, MG, Brasil.; 4 Department of Basic and Clinical Translational Sciences Tufts University School of Dental Medicine Boston MA USA Department of Basic and Clinical Translational Sciences, Tufts University School of Dental Medicine, Boston, MA, USA.; 5 East Carolina University ECU School of Dental Medicine Greenville NC USA East Carolina University, ECU School of Dental Medicine, Greenville, NC, USA.; 6 University of Florida College of Dentistry Department of Periodontology Gainesville FL USA University of Florida College of Dentistry, Department of Periodontology, Gainesville, FL, USA.

**Keywords:** Periodontitis, Transcriptome, Gene expression and regulatory networks, Bioinformatics, RNA sequencing

## Abstract

Periodontitis involves complex immune, epithelial, and metabolic disruptions. High-throughput transcriptomic approaches can reveal key regulatory networks underpinning disease progression.

**Objective:**

To investigate the transcriptomic profile and dysregulated molecular pathways associated with severe periodontitis.

**Methodology:**

Gingival tissues from patients with severe periodontitis (*n*=11) and periodontally healthy controls (*n*=11) were compared using RNA sequencing in this cross-sectional study. Differentially expressed genes (DEGs) were identified and analyzed using Ingenuity Pathway Analysis (IPA). Selected DEGs were validated at the protein level via immunohistochemistry (IHC).

**Results:**

A total of 909 DEGs were upregulated and 742 were downregulated in periodontitis versus healthy tissues. Highly upregulated genes included MT-RNR1, MTRNR2L12, pseudogenes, long non-coding RNAs, and immunoglobulins. Downregulated genes included ADGRG7, C6orf15, members of enzyme families, keratin family members, loricrin (LOR), and immune modulators, such as CD207 (Langerin) and DEFB4A. IPA predicted the Wnt/β-catenin pathway as the most upregulated and the IL-17 signaling pathway as the most suppressed canonical pathway in periodontitis. The expression of the level of protein of LOR, Wnt10b, JUN, and FOS was confirmed by IHC.

**Conclusion:**

Dysregulation in key canonical signaling pathways and the altered expression of genes critical to cell chemotaxis, innate immunity, and epithelial barrier integrity seem to play a pivotal role in the pathogenesis of periodontitis.

## Graphical abstract

**Figure:**
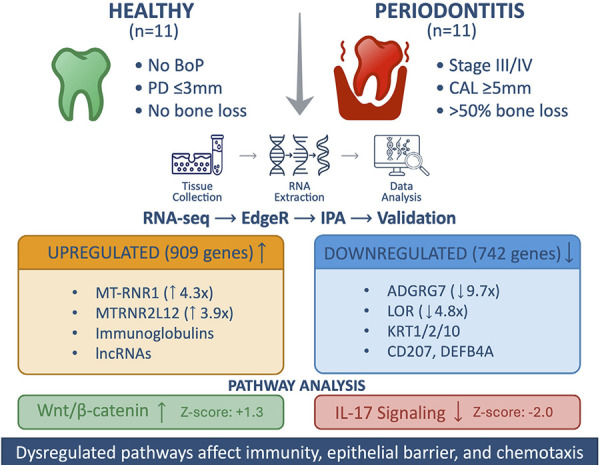

## Introduction

Periodontitis is a highly prevalent oral disease characterized by subgingival biofilm dysbiosis that triggers destructive host immune-inflammatory responses, leading to clinical attachment and bone loss.^1^ There were significant technological advances in periodontal microbial identification recently, supporting preventive and therapeutic approaches primarily targeting the microbial component.^2^ However, progress in understanding the underlying immunological basis has been more limited, hindering the clinical application of host modulation therapies for periodontal disease management.

Despite challenges in unraveling precise pathogenic mechanisms, there are significant differences in immune-inflammatory status between healthy and diseased periodontal tissues at both cellular and molecular levels.^3^ Most studies regarding the molecular level have focused on specific genes and/or proteins associated with host response using single-plex, low- to mid-plex, or microarray-based techniques.^4^ While these conventional profiling technologies provide valuable data on health-disease differences, their limitations affect the comprehensive assessment of the entire immune-inflammatory component spectrum involved in periodontitis.

Immunological research has increasingly employed high-throughput approaches to improve identification of transcripts, splice variants, and non-coding transcripts (microRNA, long non-coding RNA [lncRNAs], and pseudogenes) with both pathological and protective properties.^5^ Researchers have utilized conventional RNA sequencing (RNA-seq) to compare transcriptomes of healthy and periodontitis-affected tissues.^6^ However, discrepancies among findings stem from variations in technical protocols (sample sizes, library construction methods, platform versions), sample characteristics (disease severity, biopsy sources, sample handling, RNA preservation methods), and patient demographics and medical histories. Moreover, current literature inadequately highlights the most significantly affected pathways in periodontal disease by the integration of transcriptome data with pathway analyses. Therefore, additional research is needed to advance understanding of transcriptome profiles and corresponding biological signaling pathways relevant to periodontal diseases.

This study aimed to assess the whole transcriptome of gingival biopsies from individuals with severe periodontitis compared to healthy controls, and to identify the canonical signaling pathways implicated in periodontitis using traditional RNA-seq technology in conjunction with Ingenuity Pathway Analysis (IPA).

## Methodology

### Study population

A total of 22 participants (aged 43-75) seeking dental treatment at the University of Florida (UF) between January 2020 and March 2023 were enrolled following the UF Institutional Review Board approval (IRB201901176) and written informed consent. This observational study adhered to the STROBE guidelines for reporting.

Participants were classified as periodontally healthy (*n*=11) or with periodontitis (*n*=11) based on standard periodontal diagnostic criteria. Healthy individuals showed <10% bleeding sites on probing (BoP), probing depths (PD)≤3 mm (assuming no pseudopockets),^7^ and no interproximal bone or clinical attachment loss. They underwent procedures unrelated to periodontitis (crown lengthening, distal wedge, or non-inflammatory extractions). The periodontitis group comprised patients with untreated localized or generalized Stage III or IV, Grade B periodontitis (2018 classification),^1^ requiring tooth extraction due to severe periodontal destruction, characterized by: PD and clinical attachment level (CAL)≥5 mm, BoP, mobility, and/or >50% radiographic bone loss.

Only systemically healthy patients were included to minimize confounding effects. Exclusion criteria included: smoking history, pregnancy, lactation, recent (≤6 months) use of antibiotics, anti-inflammatories, immunosuppressants, or bone metabolism modulators (hormone replacement therapy, bisphosphonates, denosumab), regular antimicrobial mouthrinse use, and any systemic conditions linked to altered periodontal progression (diabetes, osteoporosis, immunodeficiency, autoimmune or hormonal disorders).^8^

### Biopsy and clinical data collection

Demographic and clinical parameters were obtained from records. Gingival biopsies containing oral, sulcular, junctional epithelium, and underlying connective tissue were collected during surgery, and diseased tissues were harvested from periodontitis-affected sites during tooth extraction. In healthy individuals, biopsies were taken from sites without BoP, clinical attachment loss, or bone loss during procedures such as crown lengthening, distal wedge, or extractions unrelated to periodontitis. Samples were rinsed in saline solution and immediately preserved in RNAlater solution (Ambion Inc., Austin, TX, USA) at -80°C until RNA extraction.

### RNA extraction

Total RNA was extracted using the TRIzol™ Reagent (Thermo Fisher Scientific, Waltham, MA, USA) following the instructions of the manufacturer. RNA was resuspended in diethylpyrocarbonate (DEPC)-treated water and stored at -80°C. RNA concentrations were determined using a Nanodrop 1000 micro-volume spectrophotometer (Nanodrop Technologies LLC, Wilmington, NC, USA). All samples were treated with DNase (Turbo DNA-free, Ambion Inc., Austin, TX, USA) before downstream analysis.

### Illumina sequencing library construction

RNA samples were quantified using the QUBIT fluorescent method (Invitrogen, Waltham, MA, USA), and quality-assessed with the Agilent Bioanalyzer (Agilent, Santa Clara, CA, USA). For each sample, 500 ng of protein-free RNA was used for library construction using the NEBNext rRNA depletion kit (#E6350) and Ultra Directional RNA Library Prep Kit for Illumina (New England Biolabs, Ipswich, MA, USA).

Two microliters of probe hybridization buffer were added to the RNA samples, followed by RNase H and DNAase I digestion. RNA was purified with NEBNext RNA purification beads (#E6350), and library construction proceeded using the NEBNext Ultra II Directional Lib Prep Kit (#E7760).

RNA was fragmented in divalent cation solution at 94°C. First-strand cDNA synthesis employed reverse transcriptase and oligo(dT) primers, followed by double-stranded cDNA (ds-cDNA) synthesis using the kits 2^nd^ strand master mix. Illumina adaptors were ligated after end-repair and dA-tailing. The library underwent amplification and purification using AMPure beads (Beckman Coulter, Pasadena, CA, USA, #A63881).

Library size and concentration were evaluated using the Agilent TapeStation with DNA5000 Screen Tape (Agilent, Santa Clara, CA, USA). Libraries showed a broad peak from 200-1000 bp, with peak intensity around 500 bp. Functionality was validated by quantitative PCR (qPCR) using the KAPA Library Quantification Kit (Kapa Biosystems, Wilmington, MA, USA, #KK4824) on a BioRad CFX 96 real-time PCR system (Bio-Rad Laboratories, Hercules, CA, USA). Barcoded libraries were pooled equimolarly. RNA-seq library construction was conducted at the Interdisciplinary Center for Biotechnology Research (ICBR) Gene Expression & Genotyping Core Facility, UF (RRID: SCR_019145).

### Illumina NovaSeq6000 sequencing

Sequencing was conducted at the ICBR NextGen DNA Sequencing Core Facility, UF (RRID: SCR_019152). Libraries were treated with Illumina Free Adapter Blocking (FAB) Reagent (#20024145) to minimize adaptor-dimers and index hopping. The library pool was diluted to 0.8 nM and sequenced on an Illumina NovaSeq6000 S4 flow cell lane (2×150 cycles) using NovaSeq Control Software v1.6 with cluster/SBS consumables v1.5. Final loading concentration was 120 pM with a 1% PhiX spike-in. The run yielded ∼2.5 billion paired end reads (∼750 Gb) with an average of Q30≥92.5% and 85.4% cluster pass filter (PF) rate. FastQ files were generated via BCL2fastQ in the Illumina BaseSpace, with an average of 75 million demultiplexed paired end reads per sample for subsequent analysis.

### RNA-Sequence quality control

RNA-seq data quality was initially evaluated using FastQC (http://www.bioinformatics.babraham.ac.uk/projects/fastqc/). Low-quality sequences were trimmed and filtered using Trimmomatic.^9^

### Analysis of differential gene expression

Analyses were conducted at the UF-ICBR Bioinformatics core. High-quality single-end reads were mapped to the GRCh38 genome assembly (https://useast.ensembl.org/Homo_sapiens/Info/Index/), using Star Aligner.^10^ Gene expression was quantified via RNA-seq by Expectation Maximization (RSEM),^11^ extracting expected read counts and fragments per kilobase of transcript per million mapped reads (FPKM) values for downstream analysis.

EdgeR^12^ was employed for differential gene expression analysis. Differentially expressed genes (DEGs) between Periodontitis and Healthy groups were identified using thresholds of false discovery rate (FDR)<0.05 and log_2_ fold change (FC)≥1. Principal component analysis (PCA) was conducted prior to the differential expression analysis; there were no outlier samples.

### Ingenuity pathway analysis (IPA)

Pathway analysis was conducted using IPA (Qiagen, Redwood City, CA). DEGs from periodontitis versus healthy tissues, with corresponding fold changes, were analyzed to interpret functional significance via Canonical Pathways (CP).

Significance threshold was set at −log(*p*-value)>1.5, with activation defined by Z-score>1 and inhibition by Z-score<-1 for Ingenuity Canonical Pathway (ICP). IPA also facilitated upstream regulatory element analysis. Disease and Biofunction analyses identified altered functional categories associated with periodontitis-related DEGs.

### Validation of DEGs by immunohistochemistry (IHC)

IHC was performed on four biologically relevant pathways observed in diseased tissues DEGs (LOR, Wnt10b, JUN, and FOS) in an independent set of four tissues per group to validate transcriptome findings at the protein level. Gingival samples were fixed in 10% neutral-buffered formalin, and 4-μm-thick paraffin-embedded sections were mounted on positively charged glass slides. All staining was conducted using a Leica RX Autostainer (Leica Biosystems, Wetzlar, Germany).

Slides underwent deparaffinization, rehydration, and heat-induced antigen retrieval using ER1 or ER2 buffers (20 min), then endogenous peroxidases were blocked with hydrogen peroxide (5 min). Tissue sections were incubated (30 min) with primary antibodies: FOS (1:500, monoclonal, 66590-1-Ig, Proteintech, Rosemont, IL, USA), JUN (1:1200, monoclonal, 66313-1-Ig, Proteintech), LOR (1:750, polyclonal, ab85679, Abcam, Inc., Cambridge, CB2 0AX, UK), and Wnt10b (1:100, polyclonal, ab217632, Abcam), then treated (8 min) with an HRP Polymer (Leica Biosystems, Wetzlar, Germany).

Specific antibody reactions were visualized using 3,3’-diaminobenzidine (DAB) (10 min), counter-stained with hematoxylin (Leica Biosystems), dehydrated, cleared, and mounted using Cytoseal media (Richard-Allan Scientific, San Diego, CA, USA). Negative controls omitted primary antibodies. Results describe cellular immunopositivity, staining patterns (membrane, cytoplasmic, nuclear), and intensity (weak, moderate, strong).

### Statistical analysis of clinical and demographic data

Clinical data were assessed for normality (Shapiro-Wilk test). Parametric methods were applied following confirmation of normal distribution. Between-group differences in age and clinical parameters were evaluated by independent t-test, while sex distribution was analyzed by Chi-square test. All analyses used a 5% significance level. No missing data were encountered in this study and no subgroup analyses, interaction testing, or sensitivity analyses were performed.

## Results

All approached people who met the inclusion criteria agreed to participate, with no exclusions due to non-participation. A total of 22 patients (11 per group) were included in the final analysis. Mean age and sex distribution did not differ significantly between the Healthy and Periodontitis groups (*p*>0.05). As expected, periodontal clinical parameters at full-mouth and sampled teeth were significantly higher in Periodontitis than in Healthy (Table 1; *p*<0.05).

**Table 1:** Age and periodontal parameters of the study population.

Parameters	Groups	
	**Healthy (*n*=11)**	**Periodontitis (*n*=11)**
Age (years)	58.4±6.8	56.7±5.2
Full Mouth % of sites with plaque	16.2±1.3	47.8±20.1*
Full Mouth % of sites with BoP	6.7±0.7	42.7±17.8*
Full Mouth PD (mm)	2.0±0.3	3.7±0.5*
Full Mouth CAL (mm)	2.2±0.2	5.2±1.1*
Sampled teeth PD (mm)	2.3±0.2	6.2±1.2*
Sampled teeth CAL (mm)	2.3±0.2	8.4±1.8*

Note: The values are presented shown as means ± standard deviations (SDs).

*Significant differences between groups (t-test; *p*<0.05).

Abbreviations: BoP, bleeding on probing; PD, probing depth; CAL, clinical attachment level.

### DEG findings

A total of 16,164 distinct mRNA transcripts were detected across all tissue samples. Among these, 1,214 were exclusively expressed in periodontitis-affected tissue, whereas 1,423 genes were uniquely expressed in healthy gingival tissues. Additionally, 1,651 genes (Supplementary Table 1) showed significant differential expression between the groups, with 909 genes upregulated and 742 genes downregulated in periodontitis-affected tissues relative to healthy tissues.

**Figure 1:**
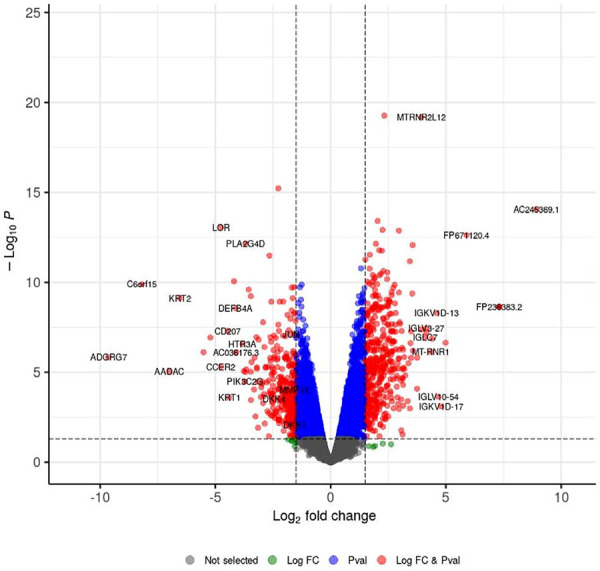
Volcano plot of differential gene expression between periodontitis and healthy groups. The x-axis represents log2 fold change, and the y-axis shows –log10(*p*-value). Vertical dashed lines indicate fold change thresholds, and the horizontal dashed line marks the significance cutoff (*p*<0.05). Red dots indicate genes with *p*-values < 0.05, upregulated on the right side of the plot and downregulated on the left. Gray dots represent genes that have not significantly changed. Green dots indicate genes meeting the log2 fold change threshold only, and blue dots indicate genes meeting the *p*-value threshold only.

Table 2 shows the top 20 upregulated and DEGs in periodontitis tissue. Among the most highly upregulated genes in periodontitis were MT-RNR1 (Log_2_FC=4.3), MTRNR2L12 (Log_2_FC=3.9), pseudogenes, lncRNAs, and immunoglobulins (Ig). ADGRG7 (Log_2_FC=-9.7) and Chromosome 6 Open Reading Frame 15 (C6orf15) (Log_2_FC=-8.2) showed the strongest downregulation in periodontitis. Additionally, members of several enzyme families were significantly downregulated, including arylacetamide deacetylase (AADAC), tyrosinase-related proteins 1 and 2 (TYRP1 and DCT), nitric oxide synthase 1 (NOS1), phospholipase A2 group IVD (PLA2G4D), and phosphatidylinositol-4-phosphate 3-kinase catalytic subunit type 2 gamma (PIK3C2G). Genes related to keratinocyte differentiation and the epidermal differentiation complex, including keratin family members (KRT1, KRT2, and KRT10) and loricrin (LOR), were also among the most downregulated genes in periodontitis. Furthermore, genes encoding proteins with critical roles in immune response, such as CD207 (Langerin) and defensin Beta 4 (DEFB4A), were significantly downregulated in periodontitis-affected tissues. Figure 1 shows the volcano plot of differential gene expression between periodontitis and healthy groups, highlighting significant up- and downregulated genes.

**Table 2:** Top 20 upregulated and downregulated DEGs in periodontitis compared to healthy tissues.

Upregulated				Downregulated			
Gene	FDR	Adjusted *p*-value	Log_**2**_FC	Gene	FDR	Adjusted *p*-value	Log_**2**_FC
AC245369.1 IGHV2-70D	9.5e-12	8.4e-15	8.92	ADGRG7	6.1e-05	1.5e-06	-9.65
FP236383.2	3.5e-07	2.24e-09	7.31	C6orf15	4.2e-08	1.3e-10	-8.18
FP671120.3	3.5e-07	2.2e-09	7.31	AADAC	0.0002	9.1e-06	-7.01
FP236383.3	3.5e-07	2.24e-09	7.31	KRT2	1.6e-07	7.6e-10	-6.53
FP671120.4	1.9e-10	2.4e-13	5.89	STAC2	3.6e-05	7.7e-07	-5.51
IGHV3-43	1.4e-05	2.3e-07	4.98	TYRP1	8.2e-06	1.2e-07	-5.21
IGKV1D-17	0.007	0.0008	4.79	LOR	8.2e-11	8.7e-14	-4.76
IGLV10-54	0.003	0.0002	4.67	CCER2	0.0001	4.9e-06	-4.76
IGKV1D-13	6.5e-07	5.2e-09	4.60	CD207 (Langerin)	4.2e-06	5.0e-08	-4.46
IGKV5-2	9.1e-06	1.3e-07	4.39	KRT1	0.003	0.0003	-4.40
MTRNR1	3.5e-05	7.4e-07	4.34	DEFB4A	4.1e-07	2.8e-09	-4.12
IGLV3-27	3.2e-06	3.5e-08	4.15	AC036176.3 Lnc-SERPINB12-3	3.6e-05	7.6e-07	-4.09
IGHV6-1	4.7e-06	6.0e-08	4.14	HTR3A	1.5e-05	2.6e-07	-3.83
IGLC7	7.8e-06	1.0 e-07	4.09	DCT	0.0002	8.5e-06	-3.76
MTRNR2L12	1.8e-16	6.7e-20	3.93	PIK3C2G	0.0006	3.3e-05	-3.72
IGHV3-49	3.7e-06	4.1e-08	3.91	SLURP1	0.0003	9.7e-06	-3.70
IGHV2-70	6.3 e-05	1.5e-06	3.75	PLA2G4D	5.01e-10	6.8e-13	-3.69
MTND2P28	0.001	8.3e-05	3.75	NOS1	0.0002	7.0e-06	-3.66
IGLC3	3.1 e-05	6.2e-07	3.57	KRT10	7.0e-08	2.5e-10	-3.55
IGLV3-1	5.7e-10	8.5e-13	3.55	NEFL	1.3e-07	5.8e-10	-3.46

Note: Adjusted *p*-value indicates probability value from statistical testing.

Abbreviations: DEGs, differentially expressed genes; FDR, false discovery rate (adjusted *p*-value for multiple testing); Log_2_FC, log_2_ fold change; ADGRG7, adhesion G protein-coupled receptor G7; C6orf15, chromosome 6 open reading frame 15; AADAC, arylacetamide deacetylase; KRT, keratin; STAC2, SH3 and cysteine-rich domain 2; TYRP1, tyrosinase-related protein 1; LOR, loricrin; CCER2, coiled-coil glutamate-rich protein 2; CD207, Langerin; DEFB4A, defensin beta 4A; DCT, dopachrome tautomerase; PIK3C2G, phosphatidylinositol-4-phosphate 3-kinase catalytic subunit type 2 gamma; SLURP1, secreted LY6/PLAUR domain containing 1; PLA2G4D, phospholipase A2 group IVD; NOS1, nitric oxide synthase 1; NEFL, neurofilament light.

### IPA

**Figure 2:**
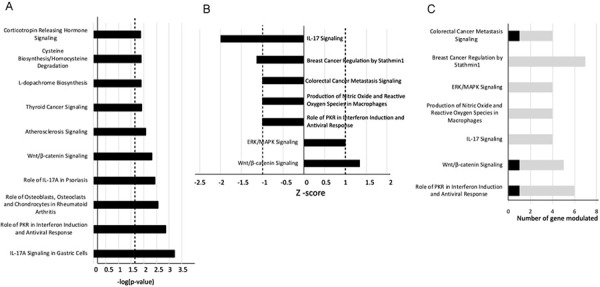
IPA analysis of the canonical pathways related to the DEGs in tissues with periodontitis compared with tissues with periodontal health. (A) The top ten dysregulated canonical signaling pathways are shown in descending order of statistical significance of −log(*p*-values). (B) The canonical pathways that were predicted to be modulated in periodontitis, as defined by a −log(*p*-value) >1.5 and Z-score ≥1 (activated) or ≤−1 (inhibited). (C) The number of upregulated and downregulated DEGs involved in each of the canonical pathways.

Overall, 25 ICPs were identified using a threshold of −log(*p*-value)>1.5. The complete list of ICPs and their associated genes is provided in Supplementary Table 2. Figure 2A displays the top ten significant CPs associated with DEGs in periodontitis, ranked by the −log(*p*-value). The highest-ranking CPs were as follows:

- IL-17A Signaling in Gastric Cells (−log(*p*-value)=3.22; overlap 11.5%:3/26)
- Role of Protein Kinase (PKR) in Interferon Induction and Antiviral Response (−log(*p*-value)=2.88; overlap 4%:5/126)
- Role of Osteoblasts, Osteoclasts, and Chondrocytes in Rheumatoid Arthritis (−log (*p*-value)=2.57; overlap 2.8%:6/216)
- Role of IL-17A in Psoriasis (−log(*p*-value)=2.45; overlap 14.3%:2/14)
- Wnt/ β-catenin Signaling (−log(*p* -value)=2.33; overlap 3%:5/169).

Applying Z-scores ≥1 and ≤-1, seven pathways showed stronger associations with periodontitis. Wnt/β-catenin Signaling (Z-score=1.3; one upregulated and four downregulated genes) and Extracellular-regulated Kinase/Mitogen-Activated Protein Kinase (ERK/MAPK) Signaling (Z-score=1; four downregulated genes) were predicted to be upregulated in periodontitis. Five pathways were predicted to be downregulated in periodontitis: IL-17 Signaling (Z-score=-2; four downregulated genes), Breast Cancer Regulation by Stathmin1 (Z-score=-1.13; seven downregulated genes), Colorectal Cancer Metastasis Signaling (Z-score=-1; one upregulated and three downregulated genes), Production of Nitric Oxide and Reactive Oxygen Species in Macrophages (Z-score=-1; four downregulated genes), and Role of PKR in Interferon Induction and Antiviral Response (Z-score=-1; one upregulated and five downregulated genes) (Figures 2B and 2C).

In the upregulated Wnt/β-catenin Signaling pathway, Wnt Family Member 10B (Wnt10B) was upregulated in periodontitis (Log_2_FC>1.5). Conversely, Dickkopf Wnt Signaling Pathway Inhibitors (DKK1 and DKK4), activator protein-1 (AP-1) transcription factor subunits FOS and JUN, and Protein Phosphatase 2 Regulatory Subunit B gamma (PPP2R2C) were downregulated (Log_2_FC<-1). In the IL-17 Signaling pathway, identified as the most suppressed CP, FOS, JUN, Matrix Metallopeptidase 13 (MMP-13), and C-C Motif Chemokine Ligand 20 (CCL20) were downregulated in periodontitis tissues (Log_2_FC<-1.5) (Supplementary Table 1 and Table 2).

Table 3 shows the five significantly enriched items in the categories of “diseases and disorders”, “molecular and cellular functions”, and “physiological system development and function” from the DEGs analysis. Upstream regulator analysis predicted significant activation of EPCAM (*p*-value=1.76E-08), whereas IL1A (*p*-value=1.12E-06), F7 (*p*-value=1.45E-05), and ERK (*p*-value =1.89E-05) were predicted to be inhibited. The ‘Neurological Disease, Organismal Injury and Abnormalities, Inflammatory Response’ (Score=21) was the most enriched network of DEGs in periodontitis.

**Table 3:** Top diseases and biological functions most significantly associated with the DEGs in periodontitis.

Top Diseases and Bio functions	*p*-value range	#molecules
Diseases and disorders		
Neurological Disease	4.84E-02 – 2.31E-07	31
Organismal Injury and Abnormalities	4.99E-02 – 2.31E-07	116
Dermatological Diseases and Conditions	3.79E-02 – 8.65E-05	26
Infectious Diseases	3.79E-02 – 1.41E-04	16
Cancer	4.99E-02 – 2.43E-04	116
Molecular and cellular functions		
Cellular Movement	4.40E-02 – 7.66E-07	13
Cellular Death and Survival	4.43E-02 – 4.09E-05	15
Cellular Function and Maintenance	1.91E-02 – 4.09E-05	10
Cellular Cycle	6.41E-03 – 1.33E-03	3
Gene Expression	3.74E-02 – 1.33E-03	13
System development and function		
Embryonic Development	4.40E-02 – 7.66E-07	7
Behavior	3.24E-02 – 2.58E-03	3
Cardiovascular System Development and Function	4.40E-02 – 6.41E-03	8
Digestive System Development and Function	6.41E-03 – 6.41E-03	1
Endocrine System Development and Function	1.91E-02 – 6.41E-03	1

Note: Results obtained from Ingenuity Pathway Analysis (IPA) of differentially expressed genes (DEGs). The *p*-value range indicates the range of significance levels for the associations between the molecules in the dataset and the functions/diseases. The “#molecules” indicates the number of molecules from the dataset that are associated with the listed function/disease.

### IHC

**Figure 3:**
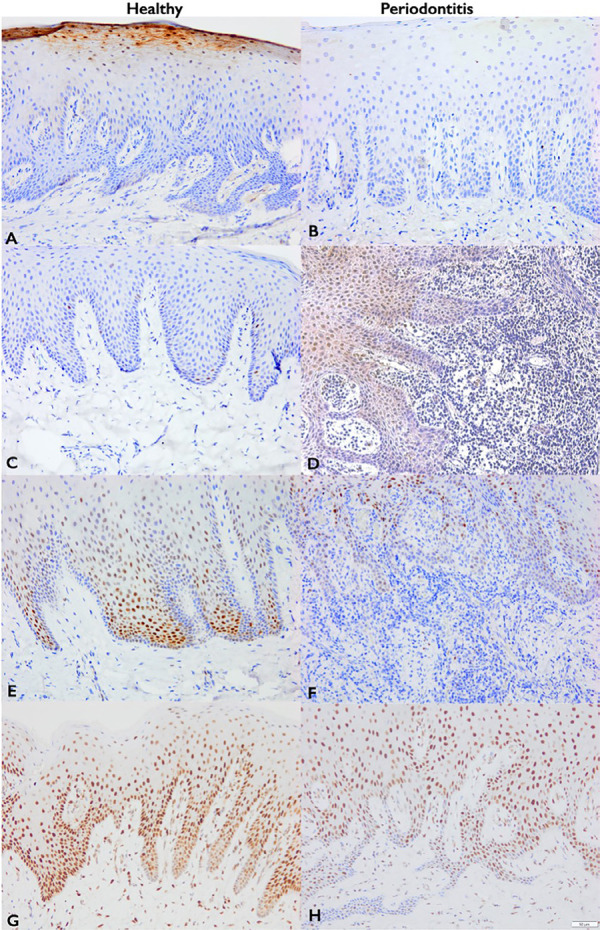
The immunohistochemical staining using antibodies against LOR (A-B), Wnt10b (C-D), FOS (E-F), and JUN (G-H), and in representative gingival biopsies from the healthy and periodontitis groups. Bar length = 50 μm, original magnification × 200.

Figure 3 shows IHC findings, in which the healthy group (Figure 3A) showed higher cytoplasmic and nuclear LOR expression in the granular layer compared to the periodontitis group (Figure 3B). The Periodontitis group showed increased nuclear Wnt10B expression in the epithelium (mainly in the suprabasal cell layer) and connective tissue (Figure 3D) compared to the Healthy group (Figure 3C). The Healthy group showed significantly higher nuclear FOS expression in the suprabasal layer of the epithelium (Figure 3E) than the Periodontitis group (Figure 3F). Moreover, the Healthy group (Figure 3G) showed higher nuclear JUN expression in the basal and suprabasal layers and connective tissue compared to the Periodontitis group (Figure 3H).

## Discussion

This study employed RNA-seq to comprehensively evaluate transcriptomic signatures of periodontitis in humans. DEGs related to inflammation, immunity, healing, metabolism, and oxidative stress emerged as potential key mediators, along with several Ingenuity Canonical Pathways significantly enriched in diseased tissues.

Consistent with previous transcriptomic studies, our analysis identified significant upregulation (log_2_FC>3.0) of immunoglobulin-encoding genes in periodontitis, reflecting the characteristic inflammatory and adaptive immune responses associated with chronic periodontitis.^13,14^ Notably, mitochondrial-derived peptides (MDPs)—specifically MOTS-c and a humanin isoform encoded by MT-RNR1 and MT-RNR2L12, respectively—were among the top 20 upregulated genes. MOTS-c regulates insulin sensitivity, diabetes,^15^ and endothelial function^16^ while also inhibiting osteoclastogenesis via osteocyte RANKL/OPG secretion and suppressing inflammation by inhibiting NF-κB and STAT1 pathways.^17^ Humanin-like proteins modulate inflammation, autophagy, cellular metabolism, and oxidative stress, exerting cytoprotective and anti-apoptotic effects in inflammatory diseases.^18^ The influence of these peptides on periodontitis needs further investigation.

Our findings on the most significantly downregulated DEGs align with previous transcriptomic studies of periodontal tissues. Liu, et al.^19^ (2020) first observed PIK3C2G, AADAC, LOR, and TYRP1 among the top ten downregulated mRNAs in periodontitis. Subsequently, Jeon, et al.^14^ (2021) reported marked suppression of LOR, KRT1, KRT2, KRT10, C6orf15, SLURP1, PLA2G4D, and DEFB4A using two independent microarray datasets. Lin, et al.^20^ (2021) further confirmed significant downregulation of AADAC, KRT1, KRT2, KRT10, LOR, and additional genes involved in epithelial function, including the calcium channel regulator STAC2 and NOS1. Gao, et al.^13^ (2022) recently identified KRT2, LOR, NEFL, and AADAC among their top 20 downregulated genes, reinforcing earlier observations. These convergent findings reveal a consistent pattern of transcriptional repression affecting genes that are essential for epithelial differentiation, structural integrity, and cellular signaling. The reproducible downregulation of keratin family members (KRT1, KRT2, KRT10), epithelial differentiation markers (LOR, SLURP1), and barrier function regulators across multiple independent studies strongly implicates epithelial barrier dysfunction as a fundamental component in periodontitis pathogenesis.

Importantly, several downregulated genes (KRT1, KRT2, KRT10, LOR, and SLURP1) are essential in barrier integrity and epithelial differentiation.^21^ Expression of epidermal differentiation complex members varies across junctional, pocket, sulcular, and external oral epithelia. Cytokeratin 1 and 10 are absent in junctional and pocket epithelia but are present in oral sulcular and external oral epithelia.^22^ SLURP1 is expressed in oral keratinocytes, periodontal ligament, and gingival fibroblasts, exerting anti-apoptotic effects beyond its role in epidermal differentiation.^23^ LOR is commonly detected in keratinized but not in non-keratinized oral epithelium.^24^ Our study confirmed LOR downregulation at the protein level (Figure 3). We hypothesize that gingival epithelial resilience against bacterial infection may diminish in periodontitis due to the downregulation of genes encoding proteins necessary for epithelial barrier function and repair. Future research should investigate these genes across different gingival histological structures to elucidate their roles in periodontitis pathogenesis.

The downregulation (log_2_FC<4.0) of immunity-related genes, including Langerin/CD207 and DEFB4A, is also notable. Langerin/CD207, which is a marker for Langerhans cells (LCs), is fundamental for T cell activation and both adaptive and innate immunity,^25^ and its downregulation may indicate a reduced LC number and impaired pathogen recognition during chronic periodontitis. Langerin/CD207 downregulation coincided with LOR downregulation, which affects both epidermal terminal differentiation and LC differentiation/maturation, impacting immune effector functions.^26^ DEFB4A encodes β-defensin peptides with antimicrobial activities and immune cell activation properties, and its downregulation, which is consistent with findings by Jeon, et al.^14^ (2021), suggests compromised or obscured protective functions during the chronic disease. Neither Langerin nor DEFB4A proteins were detected by IHC in either of our samples.

LncRNAs have emerged as important gene expression regulators beyond mRNA stability and translation in periodontitis.^20^ Our study found FP236383.2, FP671120.3, FP236383.3, and FP671120.4 among the top five upregulated DEGs. Further research is required to validate their involvement in periodontitis pathogenesis.

IPA analysis predicted significant activation of Wnt/β-catenin signaling in periodontitis. This pathway modulates osteogenesis, cell proliferation, apoptosis, inflammation, and immunity,^27^ playing either anti-inflammatory or pro-inflammatory roles depending on pathogenic stimulus, cell type, and interactions with other signaling pathways. Wnt/β-catenin pathway activation can downregulate pro-inflammatory responses as a compensatory mechanism, while pro-inflammatory stimuli can increase expression of Wnt/β-catenin-related molecules. This suggests that β-catenin activity is essential for chronic inflammation,^28^ as observed in periodontitis. In this study, Wnt/β-catenin pathway activation was driven by an increased WNT10B expression alongside decreased DKK1, DKK4, JUN, and PPP2R2C expression. WNT10B, upregulated at mRNA and protein levels, specifically activates canonical Wnt/β-catenin signaling. Downregulation of DKK1 and DKK4, Wnt signaling antagonists, further indicates pathway activation. The β-catenin-independent noncanonical Wnt/planar cell polarity (PCP) pathway induces c-Jun N-terminal kinase (JNK) activation,^29^ thus c-JUN downregulation may suggest Wnt/PCP pathway inhibition, reinforcing Wnt/β-catenin pathway activation via antagonistic interactions.^29^ These findings warrant further investigation into Wnt/β-catenin pathway involvement in periodontitis.

Consistent with our results, previous analyses suggest FOS and JUN downregulation in periodontitis.^30^ c-FOS and c-JUN, validated at the protein level, are components of the dimeric transcription factor AP-1, consisting primarily of FOS proteins (c-FOS, FOSB, FRA-1, and FRA-2, encoded by FOSL1 and FOSL2) and JUN proteins (c-JUN, JUNB, and JUND).^31^ These factors regulate cell growth, division, apoptosis, migration, inflammation, and bone turnover. Additional genes encoding FOS and JUN family members (JUNB, FOSB, FOSL1) were significantly downregulated in periodontitis (Supplementary Table 1). AP-1 regulates MMPs,^31^ with c-FOS and c-JUN heterodimers specifically regulating MMP13,^32^ possibly through the transcription factor ATF3 activation.^33^ Accordingly, downregulation of JUN and FOS family members coincided with decreased MMP13 and ATF3 expression (Supplementary Table 1), suggesting reduced AP-1-mediated process during chronic periodontitis. Further studies are needed to elucidate AP-1 complex involvement in periodontitis.

The IL-17 signaling pathway, which induces inflammation to counteract pathogens,^34^ was intriguingly downregulated in periodontitis. IPA identified four downregulated DEGs directly associated with the IL-17 signaling (CCL20, FOS, JUN, MMP13), while IL-17RE was also downregulated (Supplementary Table 1). The IL-17 family comprises six cytokines (IL-17A to IL-17F) and five receptors (IL-17RA to IL-17RE). IL-17A is the most extensively studied IL-17 cytokine in periodontitis, followed by IL-17F.^35^ IL-17RE specifically binds IL-17C, influencing Th17 cell function and enhancing adaptive immunity against pathogens.^36^ This is also essential in host mucosal defense against infection by enhancing innate barriers.^36^ IL-17C stimulates expression of epithelial host defense mechanisms, including hBD2 (DEFB4A), S100A8, CCL20, and the CXCL family,^37^ all of which were downregulated in our study (Supplementary Table 1). CCL20 contributes to innate immunity and Th17 cells recruitment,^38^ with its dysregulation weakening mucosal structures and impairing immunity.^39^ The impact of IL-17 signaling downregulation on periodontitis represents an interesting research area.

The main strength of this study lies in the use of IPA to identify signaling pathways associated with DEGs. However, limitations include its exploratory and cross-sectional design, and the mixed cell population (epithelial, inflammatory, endothelial cells, and fibroblasts) in the transcriptional profile. Further studies should clarify profiles of individual cell types. Despite the small sample size, a previous study by Ching, Huang and Garmire^40^ (2014) supports using a minimum of five samples per group, demonstrating high performance of edgeR with deep sequencing per sample, enhancing study power.^40^

## Conclusion

Periodontitis is characterized by dysregulation of key canonical signaling pathways, notably Wnt/β-catenin and IL-17 signaling. Altered expression of genes involved in cell chemotaxis, innate immunity, and epithelial barrier integrity likely plays fundamental roles in disease pathogenesis. Such findings, pending external validation, provide valuable insights for predicting disease onset and progression, guiding mechanistic investigations, discovering biomarkers, and developing targeted preventive and therapeutic approaches.
